# GOGO: An improved algorithm to measure the semantic similarity between gene ontology terms

**DOI:** 10.1038/s41598-018-33219-y

**Published:** 2018-10-10

**Authors:** Chenguang Zhao, Zheng Wang

**Affiliations:** 1 School of Computer Science and Computer Engineering, University of Southern Mississippi, 118 College Drive, Hattiesburg, MS 39406 USA; 2 Department of Computer Science, University of Miami, 1365 Memorial Drive, Coral Gables, FL 33124 USA

## Abstract

Measuring the semantic similarity between Gene Ontology (GO) terms is an essential step in functional bioinformatics research. We implemented a software named GOGO for calculating the semantic similarity between GO terms. GOGO has the advantages of both information-content-based and hybrid methods, such as Resnik’s and Wang’s methods. Moreover, GOGO is relatively fast and does not need to calculate information content (IC) from a large gene annotation corpus but still has the advantage of using IC. This is achieved by considering the number of children nodes in the GO directed acyclic graphs when calculating the semantic contribution of an ancestor node giving to its descendent nodes. GOGO can calculate functional similarities between genes and then cluster genes based on their functional similarities. Evaluations performed on multiple pathways retrieved from the saccharomyces genome database (SGD) show that GOGO can accurately and robustly cluster genes based on functional similarities. We release GOGO as a web server and also as a stand-alone tool, which allows convenient execution of the tool for a small number of GO terms or integration of the tool into bioinformatics pipelines for large-scale calculations. GOGO can be freely accessed or downloaded from http://dna.cs.miami.edu/GOGO/.

## Introduction

Inferring semantic similarities between Gene Ontology (GO)^1^ terms is a fundamental component in functional bioinformatics research, such as gene clustering^2–4^, protein function prediction^5,6^ and gene-gene interactions validations^7–9^. Using protein function prediction as an example, it is common that the predicted protein functions of a large number of proteins (e.g., ~100,000 proteins for CAFA2^6^) in the format of GO terms are needed to be evaluated with the GO terms obtained by experimental approaches. This process usually needs to calculate the similarities between a huge number of GO-term pairs. Therefore, an accurate and fast algorithm for calculating similarities of GO terms is essential.

Gene Ontology^1^ uses three directed acyclic graphs (DAGs) to define the functions of a gene product (such as a protein): molecular function ontology (MFO), biological process ontology (BPO), and cellular component ontology (CCO). Every node in a DAG represents a GO term; and two connected GO terms are linked by different types of edges indicating different relationships. The most commonly used relationships are “is a”, “part of”, and “regulates”. Some edges exist between DAGs of different ontologies. For example, 1,093 GO terms of MFO are “part of” the GO terms of BPO based on the GO definition released on August 11, 2018.

Methods have been developed to measure the semantic similarity between GO terms. These existing methods can be classified into edge- or path-based, information content (IC)-based, node-based, and hybrid methods. The edge-based methods measure the similarities of two GO terms based on the number of edges between them^10^, usually the number of edges along the shortest path between two GO terms. For example, Wu & Palme^11^ used the common path from the lowest common ancestor node of two GO terms to define semantic similarity. However, the edge-based approaches are not in favour because edges with the same depth in the DAG may not have the same semantic distance; and the edges are usually not uniformly distributed in the DAGs^12^.

Node-based methods use the properties of the query nodes and their ancestor or descendant nodes to indicate similarities, which represent the most popular direction in this area. Resnik uses the IC of the most informative common ancestor (MICA) of two GO terms as the semantic similarity^13^. The lowest common ancestor node and the MICA refer to the same ancestor of two GO terms. The former is presented in the context of searching common path between GO terms, whereas the latter is presented in the context of IC of GO terms. Jiang and Conrath’s^14^ method and Lin’s^15^ method consider the IC values of the two query GO terms when calculating their semantic similarity. Schlicker *et al*. proposed the relevance similarity measure^16^, which reflected the location of the query GO terms in the DAG by considering the properties of MICA^17^. Li *et al*.^18^ introduced a new concept called information coefficient based on Lin’s method to integrate DAG information of query terms into calculation. Mazandu and Mulder have released Nunivers^19^, a method that normalizes the IC-based semantic similarity to 1 when measuring the similarity between the same GO terms. To avoid over-reliance on MICA, Couto *et al*. designed GraSM that could be applied to any IC-based methods, in which the semantic similarity was calculated by the average IC of the disjunctive common ancestors (DCAs) instead of MICA. Moreover, Couto and Silva have implemented DiShIn, which identifies DCA by the number of distinct paths from the query GO terms to MICA^20^. To make the calculation of semantic similarity more efficient, Zhang and Lai built GraSM using the exclusively inherited shared information (EISI) that could be applied to any IC-based methods.

The IC-based methods have an obvious advantage, that is, it uses IC to indicate the specificity of a GO term, which avoids the problems of ununiform semantic distance and edge density. However, calculating IC from annotation corpora can cause problems. As reviewed by Guzzi *et al*.^21^, in a corpus, many annotations are shallow in the DAG, which are very generic terms without describing particular molecular function, biological process, or cellular component. Moreover, since the calculation of IC depends on an annotation corpus that links a large number of genes or proteins to GO terms, it has the problem that the same GO term may have different IC values when different corpora are used. Also, the IC is biased by the research trend^12^: the GO terms related to popular fields tend to be annotated more frequently than the ones related to other unpopular fields; and the annotation of some terms may not even be found in the corpus^17^. These issues largely limit the performance and usefulness of the methods that only consider information content.

To avoid the drawbacks of the IC-based approaches, many hybrid methods have been developed that consider both edge and node in the DAG. Wang *et al*.^22^ published a hybrid method that calculated the semantic similarities based on the topology of GO DAG. Wang *et al*. incorporated the concept of semantic contribution, which could be considered as the semantic impact an ancestor node gave to its descendent nodes. Calculating semantic similarities from the GO DAG instead of IC makes Wang’s method do not need to calculate the IC values in advance. It also makes Wang's method more stable than Resnik’s method because of the above-mentioned drawbacks of the IC-based methods. GO-universal^23,24^ calculates semantic similarity by measuring the topological position characteristics in the GO DAG that considers the number of children terms instead of the frequency of terms from the annotation corpus as IC does. GO-universal defines the topological position characteristic of the root to be 1 and calculates the topological position characteristic of a non-root GO term by multiplying a ratio based on the number of children of all ancestor GO terms. Nagar and Al-Mubaid designed a hybrid structural similarity method using the shortest path plus either IC generated from corpora or structure-based IC generated from DAG^25^.

The functional similarity between gene products is important in gene classification, which is usually measured by semantic similarities between the annotated GO terms of each gene. The existing methods can be grouped into two categories, namely group-wise and pair-wise methods. Group-wise methods calculate functional similarity without considering the semantic similarity between GO terms^12^. Instead, it calculates global similarity between the two gene products^12^. For example, Mistry and Pavlidis used term overlap (also called “TO”)^26^ to measure the functional similarity between two gene products, in which functional similarity was calculated as the number of common GO terms from two genes. On the other hand, pairwise methods take advantage of semantic similarities between GO terms because they can mix semantic similarities by different strategies, such as Average (Avg)^27^, Best-Match Average (BMA)^23,28^, Average Best-Matches (ABM)^22,29^, Maximum (Max)^30^, and Best Match Maximum (BMM)^16,24^.

Different approaches have been used to evaluate the inferred semantic similarities between GO terms, although the standard assessment strategy is still under debate. Guo *et al*. evaluated multiple methods’ (Resnik’s, Lin’s, Jiang and Conrath’s) abilities of characterizing human regulatory pathways, in which Resnik was found to achieve the best performance^31^. They found that pair-wise methods have a better performance than group-wise methods. Wang *et al*.^22^ demonstrated that the gene clusters generated from their method were more similar to the pathways (based on co-expression data) defined in the saccharomyces genome database (SGD)^32^. However, Wang’s method also has disadvantages in some situations compared to the IC-based approaches, which will be illustrated later in this paper. Recently, Nagar and Al-Mubaid^25^ evaluated the performances of multiple methods at classifying interacting protein pairs using confusion matrix. Specifically, they drew the Receiver Operating Characteristic (ROC) curves and calculated the area under the curve (AUC).

In this paper, we present GOGO that is also based on GO DAG topology instead of IC which means it is stable (the advantage of Wang’s method that avoids the drawbacks of using IC). Moreover, GOGO also has the advantages of IC-based methods by considering the number of children nodes. This is based on our statistical finding that the number of children of a GO term is negatively correlated with the IC value of the GO term. Moreover, GOGO can calculate functional similarities between gene pairs or among a list of genes, in which each of the genes has one or more GO terms. GOGO can also cluster multiple genes based on their functional similarities by using the affinity propagation clustering algorithm^33^.

## Results

### Correlation between information content and the number of children

**Figure 1:**
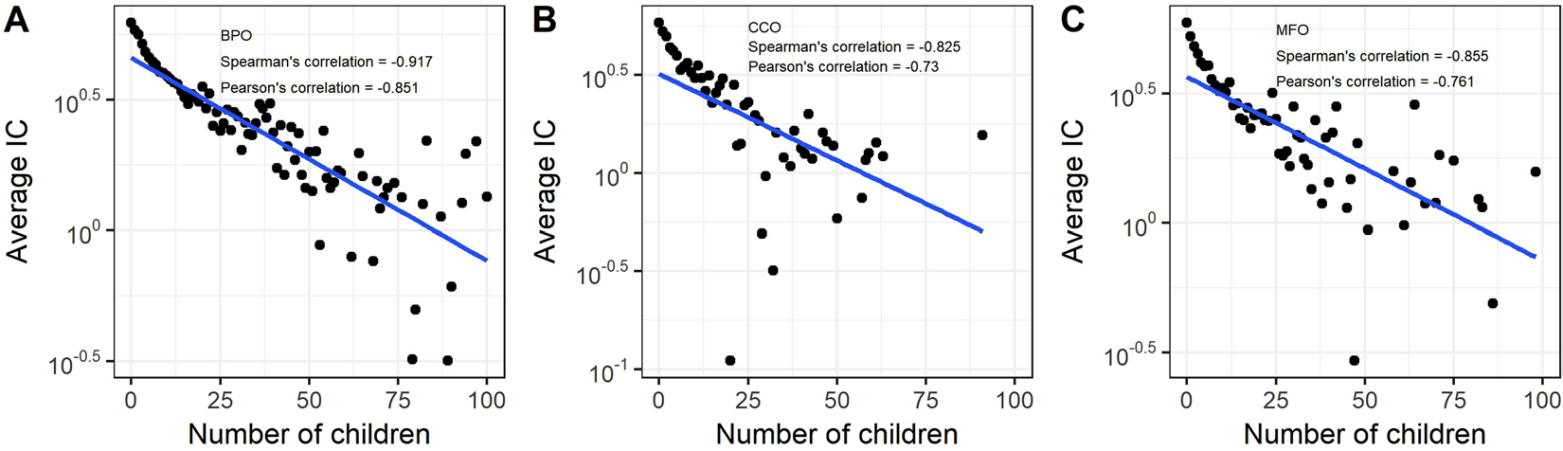
The scatter plot of the log of average IC of GO terms and the number of children nodes in GO DAG. (**A**–**C**) The plots based on BPO, CCO, and MFO, respectively. Spearman’s rank correlation and Pearson’s correlation are shown in the plots. IC was generated from the UniProt corpus including ~43 million proteins.

In Fig. 1, based on the UniProt^34^ corpus including ~43 million proteins, we plotted the relationship between the log of average IC and the number of children nodes in the GO DAG. To better illustrate the relationship, we removed some data points with extreme values, such as the number of children nodes >100 (9, 11, and 5 points removed for BPO, MFO, and CCO, respectively). These points have low average IC values that are close to zero. We found strong negative correlations between the average IC and the number of children nodes. Spearman’s rank correlation coefficients are −0.917, −0.825, and −0.855 for BPO, CCO, and MFO, respectively. Pearson’s correlation coefficients are −0.851, −0.73, and −0.761, respectively. Based on this finding, we used the number of children nodes to indicate information content in our method, which avoided calculating IC from an annotation corpus.

### Examples showing the advantage of GOGO

**Figure 2:**
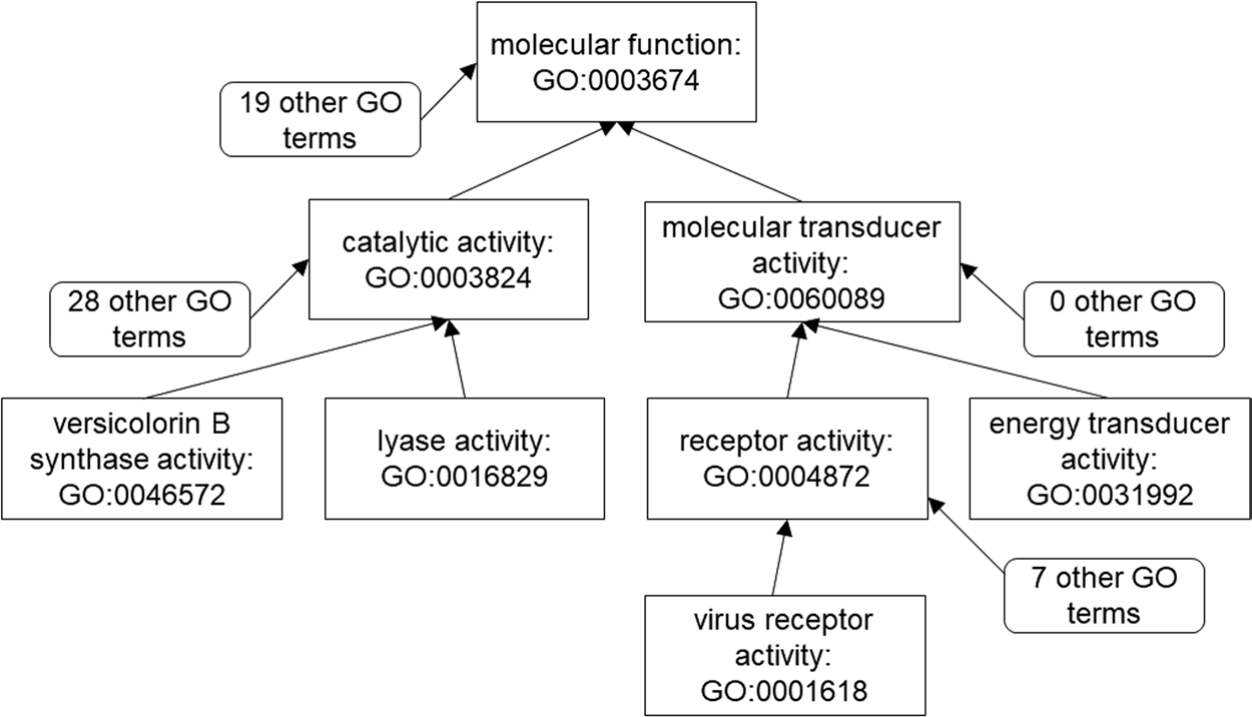
A partial GO DAG of MFO illustrating examples of calculating semantic similarities by GOGO, Wang, and IC-based methods.

Figure 2 shows a four-layer DAG containing the root node in MFO GO:0003674 and some children nodes in the first three levels below the root (based on the GO definition released on September 10, 2016). As shown in Table 1, GOGO generates 0.387 and 0.529 for GO-term pair (GO: 0046572 and GO: 0016829) and pair (GO: 0004872, GO: 0031992). IC-based methods (i.e. Resnik, Lin, Li *et al*., Relevance, Nunivers) generate different similarity values: Resnik outputs 0.075 and 0.232, whereas Wang’s method generates 0.590 for both pairs (semantic similarities of all methods except GOGO were calculated and normalized by A-Da-GO^24^ with default settings). Obviously, Wang’s method cannot distinguish these two pairs, but IC-based methods can. GOGO can also tell the difference between these two pairs by considering the number of children nodes of the ancestor nodes when calculating semantic contribution. As shown in Fig. 2, node GO:0003824 has 28 other children nodes, whereas GO:0060089 has no other children node. This makes the semantic contribution from GO:0003824 to the pair (GO: 0046572, GO: 0016829) much less than the semantic contribution from GO: 0060089 to pair (GO: 0004872, GO: 0031992). In this regard, GOGO has the advantage of IC-based methods but with no need to calculate IC, which makes the semantic similarity values stable and saves computational time.

**Table 1:** Semantic similarities between GO-term pairs in the examples shown in Fig. 2.

	Sim (0046572, 0016829)	Sim (0004872, 0031992)	Sim (0060089, 0001618)	Sim (0060089, 0004872)
Resnik	0.075	0.232	0.232	0.232
Lin	0.121	0.547	0.399	0.730
Li	0.071	0.445	0.489	0.894
Relevance	0.092	0.541	0.483	0.884
Nunivers	0.075	0.414	0.323	0.809
Wang	0.590	0.590	0.477	0.643
GOGO	0.387	0.529	0.455	0.592

IC-based methods (i.e., Resnik, Lin, Li *et al*., Relevance, and Nunivers) and hybrid method (i.e., Wang) were executed in order to compare with GOGO.

Another example is to compare pair (GO:0060089, GO:0004872) and pair (GO:0060089, GO:0001618), which are between a parent node (GO:0060089) and its child node (GO:0004872) and between a grandparent node (GO:0060089) and its grandchild node (GO:0001618). Table 1 shows that Resnik’ method fails to tell the difference of two pairs and generates the same semantic similarities. Other IC-based methods, GOGO, and Wang’s method can assign a higher similarity score to pair (GO: 0060089, GO: 0004872), the parent-children case, which is consistent with human perspectives that a parent node and its child node should be semantically closer than the grandparent node and its grandchild node.

### Comparisons between GOGO and existing methods

Table 2 shows the Pearson’s correlation coefficients between GOGO’s semantic similarities and seven popular methods including Wang’s method^22^, GO-universal^23,24^, Resnik’s method^13^, Lin’s method^15^, Li *et al*.^18^, Relevance^18^, and Nunivers^19^. For each gene ontology, the correlation matrix was generated based on randomly selected 500 GO-term pairs with semantic similarity greater or equal to 0.5 (based on Wang’s method). We set this threshold because random pairs usually have extremely low similarities that do not well represent a method’s performance. We also generated the correlation matrices based on random GO-term pairs without threshold (see Supplementary Table S1). It can be noticed that GOGO and Wang’s method have the highest correlation; and the correlations between IC-based methods are larger than 0.9 in BPO. We also found that GOGO and IC-based methods were better correlated than Wang’s and IC-based methods in BPO.

**Table 2:** The Pearson’s correlation matrices between GOGO and other methods in BPO, CCO, and MFO.

	GOGO	Wang *et al*.	Resnik	GO-universal	Lin	Li *et al*.	Nunivers	Relevance
**BPO**								
GOGO	1.00	0.77	0.42	0.42	0.49	0.50	0.46	0.49
Wang *et al*.		1.00	0.39	0.61	0.45	0.46	0.45	0.45
Resnik			1.00	0.25	0.89	0.91	0.91	0.89
GO-universal				1.00	0.24	0.25	0.26	0.24
Lin					1.00	1.00	0.96	1.00
Li *et al*.						1.00	0.97	1.00
Nunivers							1.00	0.96
Relevance								1.00
**CCO**								
GOGO	1.00	0.80	0.27	0.39	0.36	0.37	0.30	0.36
Wang *et al*.		1.00	0.44	0.71	0.38	0.40	0.38	0.38
Resnik			1.00	0.33	0.82	0.85	0.85	0.82
GO-universal				1.00	0.10	0.14	0.18	0.10
Lin					1.00	1.00	0.96	1.00
Li *et al*.						1.00	0.97	1.00
Nunivers							1.00	0.96
Relevance								1.00
**MFO**								
GOGO	1.00	0.82	0.32	0.47	0.42	0.42	0.36	0.41
Wang *et al*.		1.00	0.46	0.67	0.43	0.45	0.41	0.43
Resnik			1.00	0.34	0.85	0.89	0.87	0.86
GO-universal				1.00	0.26	0.27	0.28	0.26
Lin					1.00	1.00	0.96	1.00
Li *et al*.						1.00	0.97	1.00
Nunivers							1.00	0.96
Relevance								1.00

For each gene ontology, Pearson’s correlation was generated based on 500 randomly-selected GO-term pairs with semantic similarities (based on Wang’s method) ≥0.5.

### Comparison of semantic values of sibling terms at different depths

Table 3 illustrates the average, standard deviation, and 95% confidence interval of the semantic similarity between sibling terms at depth three and seven. For BPO, we randomly selected 200 sibling GO-term pairs at depth three and seven in GO DAG. At the relatively shallow depth, we found that semantic similarity of sibling pairs calculated by GOGO had the smallest standard deviation. As the depth increased, the standard deviation of GOGO, IC-based methods, and GO-universal significantly changed, which indicated that semantic similarities of the methods considering IC or the number of children could be affected by the depth in the GO DAG.

**Table 3:** Mean, standard deviation, and 95% confidence interval of the semantic similarity between sibling GO terms in the GO DAG of BPO at depths 3 and 7.

BPO		GOGO	Wang	Resnik	GO-universal	Lin	Li	Nunivers	Relevance
Depth = 3	Mean	0.29	0.33	0.33	0.13	0.52	0.46	0.47	0.51
	Standard deviation	0.10	0.15	0.18	0.13	0.24	0.24	0.24	0.25
	95% confidence interval	(0.281, 0.308)	(0.312, 0.353)	(0.297, 0.357)	(0.111, 0.149)	(0.484, 0.564)	(0.417, 0.496)	(0.431, 0.511)	(0.473, 0.556)
Depth = 7	Mean	0.53	0.67	0.61	0.43	0.80	0.74	0.75	0.80
	Standard deviation	0.15	0.16	0.10	0.23	0.11	0.11	0.13	0.11
	95% confidence interval	(0.506, 0.546)	(0.645, 0.69)	(0.592, 0.627)	(0.398, 0.465)	(0.779, 0.82)	(0.722, 0.763)	(0.726, 0.772	(0.779, 0.82)

The result was generated based on 200 randomly-selected GO-term pairs.

### Evaluation of GOGO by clustering genes in yeast pathways

**Figure 3:**
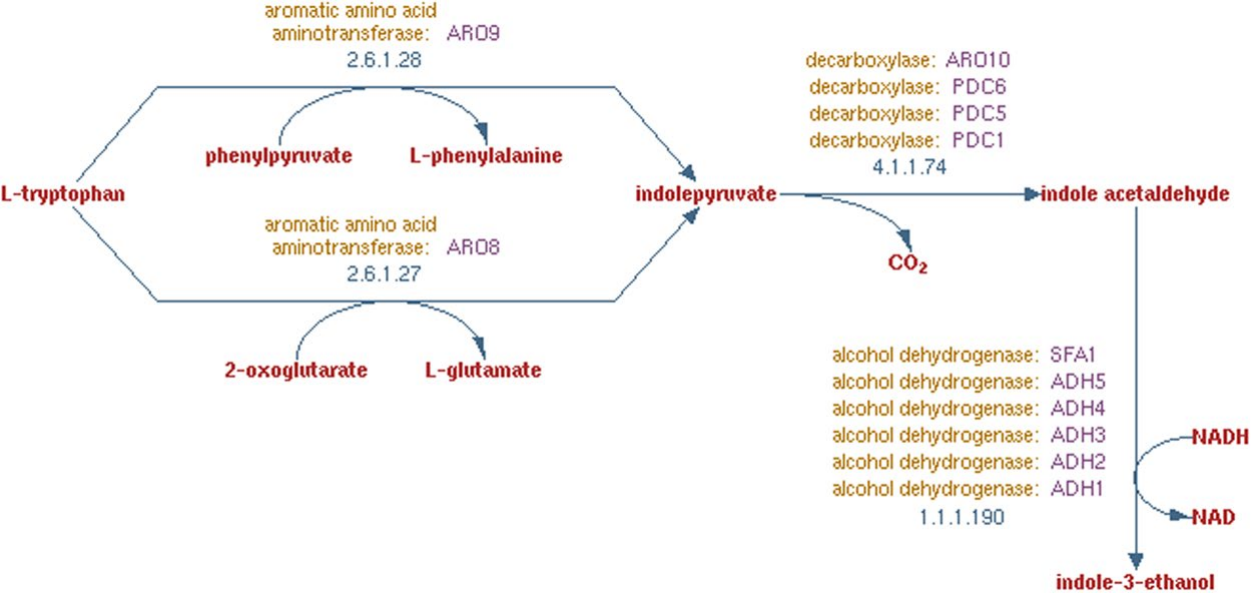
The tryptophan degradation pathway retrieved from the SGD database, in which ARO8 and ARO9 belong to the aromatic amino acid aminotransferase cluster; PDC1, PDC5, PDC6, and ARO10 belong to the decarboxylase cluster; and ADH1~5 and SFA1 belong to the alcohol dehydrogenase cluster. This Figure was made by modifying the image downloaded from the website of the SGD database.

We used six yeast biochemical pathways retrieved from the SGD^32^ to evaluate GOGO based on GO term semantic similarities. These six pathways are “tryptophan degradation”, “mevalonate pathway”, “phenylalanine degradation”, “removal of superoxide radicals”, “valine degradation”, and “mannose degradation”. The GO terms for each gene were also downloaded from the SGD database. These GO terms may be annotated with various evidence codes. In our evaluation, we only used the GO terms with experimental evidence codes including “EXP”, “IDA”, “IPI”, “IMP”, “IGI” and “IEP”, i.e., not using the GO terms with evidence codes indicating they were annotated based on e.g., computational predictions. Figure 3 shows the “tryptophan degradation” pathway; and Supplementary Figs S1–5 show the other pathways.

We tested GOGO, GOGO^regulates^ (a version of GOGO that also considers the “regulate” relationship), Wang’s, and Resnik’s methods on the same pathway “tryptophan degradation” as Wang *et al*.^22^ previously performed in their evaluations. Supplementary Tables S2–4 show the similarities between each gene pair by GOGO, Wang’s, and Resnik’s methods. Because Wang’s method used the pairwise mixing strategy ABM, in order to compare with Wang’s method we used the same mixing strategy for all other methods.

The clustering results of the pathway “tryptophan degradation” are shown in Table 4, which indicate that only the clustering results of GOGO and GOGO^regulates^ are completely consistent with the pathway retrieved from the SGD. We performed the same procedures on the other five pathways in BPO, CCO, and MFO; and we show the clustering results from GOGO and other methods in Supplementary Tables S5–9 (some genes of pathways do not have available GO terms in certain ontologies and therefore clustering results are not included). We found that GOGO and GOGO^regulates^ achieved the same performance; and the “regulates” relationships only have a small effect on functional similarities. Therefore, we only tested GOGO for the rest of the evaluations.

**Table 4:** Gene clustering results in the tryptophan degradation pathway.

	GOGO	GOGO^reguates^	Wang	Resnik	SGD
BPO Clustering Result	ADH1	ADH1	ADH1	ADH1	ADH1
	ADH2	ADH2	ADH3	ADH2	ADH2
	ADH3	ADH3	ADH5	ADH3	ADH3
	ADH4	ADH4		ADH4	ADH4
	ADH5	ADH5	ADH2	ADH5	ADH5
	SFA1	SFA1			SFA1
			ADH4	SFA1	
	PDC1	PDC1	SFA1		PDC1
	PDC5	PDC5	PDC6	PDC1	PDC5
	PDC6	PDC6	ARO10	PDC5	PDC6
	ARO10	ARO10		PDC6	ARO10
			PDC1	ARO10	
	ARO8	ARO8	PDC5		ARO8
	ARO9	ARO9		ARO8	ARO9
			ARO8	ARO9	
			ARO9		

GOGO, GOGO^regulates^, Wang, Resnik are the methods used to calculate semantic similarities. SGD indicates the true clusters based on the pathway downloaded from the SGD database.

### Testing the ability to correctly cluster genes with noises added

In the previous section, we applied semantic similarity methods only on the genes that exist in the target pathway. However, in order to test the performance of these methods when genes outside of the target pathway are added, we performed another round of evaluations. This time, we randomly selected 50% more genes (e.g., if the target pathway has 10 genes, we added 10 * 50% = 5 genes as extra input to the methods) from all other SGD pathways. We evaluated the performance using Matthew’s correlation coefficient (MCC) (for details about the evaluation procedure see “Evaluating the clustering performance using Mathew’s correlation coefficient” in “Methods”).

**Figure 4:**
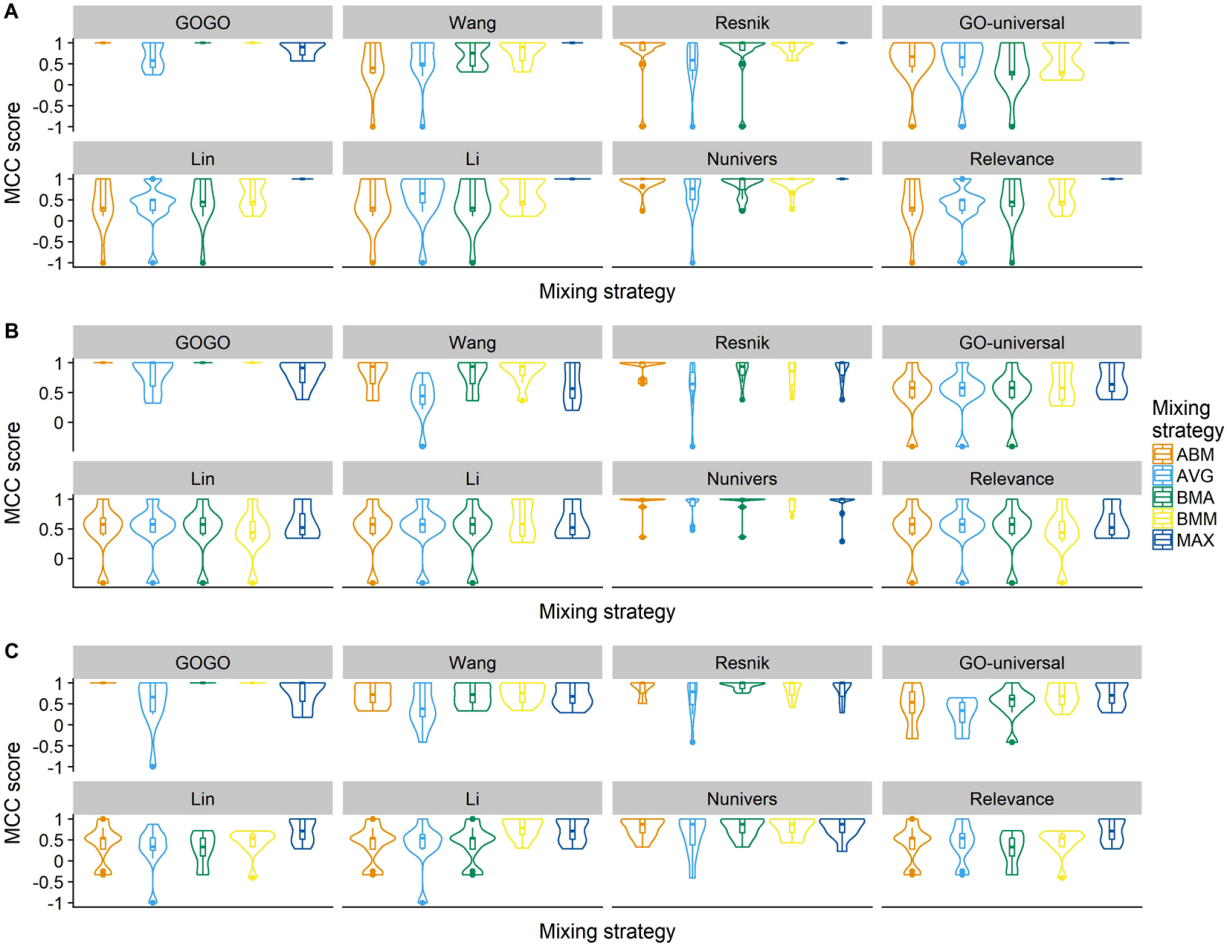
Violin and box plots of average MCC scores on pathways in BPO. (**A**) The MCC scores calculated when no outside genes were added i.e., only using the genes originally existing in the target pathways. (**B**) The MCC scores after randomly-selected outside genes were added. (**C**) The MCC scores after randomly-selected outside genes from the same EC category were added, i.e., the first two digits of EC numbers are the same.

In Fig. 4, we use violin (showing the distribution of the data) and box plot to display the MCC scores for pathways in BPO (the calculations of the other seven methods were performed by the tool A-DaGO-Fun^24^ with default settings). Figure 4A shows the MCC scores calculated by GOGO and other seven methods before adding outside genes, whereas Fig. 4B after adding outside genes. We also tested different mixing strategies in Fig. 4.

From Fig. 4A, we can find that GOGO can successfully classify genes using ABM, BMA, and BMM strategies. From Fig. 4B, we also find that the performance of other methods drops significantly when outside gene are added. However, GOGO can still maintain a good performance indicating that GOGO performs most robustly than other methods in the selected pathways.

Moreover, we also performed another evaluation for a harder configuration. For each cluster, we randomly selected 50% outside genes with top two levels of Enzyme Commission (EC) number^35^ are the same as the genes in the cluster. For example, we selected a gene with EC number starting with 2.6 as outsider gene for the cluster originally containing ARO8 (EC number 2.6.1.28) and ARO9 (EC number 2.6.1.27). Figure 4C, a stricter situation than Fig. 4B, shows that GOGO still can successfully generate correct clusters using ABM, BMA, and BMM strategies in BPO. Supplementary Figs S6,7 show the MCC scores on the same data set in CCO and MFO, in which we do not see the same good performance. The reason of this may be that the available GO terms in CCO and MFO are much less than the ones in BPO.

### Comparison of execution time

Supplementary Table S10 shows the running time of GOGO and other six popular methods based on randomly-selected 100 pairs of BPO GO terms. The running time of GOGO was obtained based on the stand-alone version of GOGO; and the other methods’ running time was based on A-DaGO-Fun^24^. Results show that the speed of GOGO is comparable with other methods. Notice that the time in Supplementary Table S10 does not include the pre-calculation of IC values for the IC-based methods, which e.g., takes ~3,781 seconds when UniProt is used as the annotation corpus.

## Methods

### Calculating IC from a large annotation corpus

The IC of a GO term is calculated as:

1 $$I{C}_{(f)}=-\,\mathrm{log}\,P(f)$$

where *P(f)* denotes the probability of the presence of the GO term *f* and its descendants. To calculate this probability, we divide the number of occurrences of GO term *f* (including its descendent GO terms) in the UniProt by the total number of occurrences of all GO terms in the same corpus.

### Semantic similarity between two GO terms

**Figure 5:**
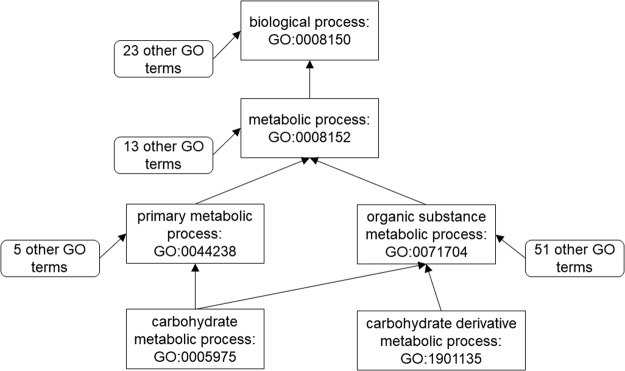
A partial GO DAG showing the ancestor nodes for GO terms carbohydrate metabolic process, GO:0005975 and carbohydrate derivative metabolic process, GO: 1901135.

We retrieved the semantic meanings and relationships between GO terms from the GO consortium^1^ released on September 10, 2016. Among all of the relationships between GO terms, the “is_a”, “part_of”, and “regulates” relationships are the most common ones. If A “is_a” B, it means that A is a subtype of B. If C is “part_of” D, it means that C and D are having a part-whole relationship. If E regulates F, it means that E directly affects the process of F. Notice that only BPO and MFO have the “regulates” relationship defined by the Gene Ontology. As for our tool GOGO, we consider “is_a” and “part_of” relationships. We also implemented another version of GOGO named GOGO^regulates^ that considers all three relationships in order to compare their performances. Figure 5 illustrates an example showing how semantic similarity between two GO terms is calculated by GOGO. It shows the GO DAG of GO:0005975, GO:1901135, and their ancestors. The arrows shown in Fig. 5 represent “is_a” relationships. For each ancestor in Fig. 5, we also show the number of children nodes.

Given a GO term A, the GO DAG of A and its ancestors are defined as ${{\rm{DAG}}}_{{\rm{A}}}=({\rm{A}},\,{{\rm{T}}}_{{\rm{A}}},\,{{\rm{E}}}_{{\rm{A}}})$, where T_A_ is the set of GO terms including A and its ancestors, and E_A_ is the set of links (i.e. edges) among nodes of T_A_ in DAG_A_. To measure the semantic contribution of ancestors to A in the GO DAG, we first calculate the weight for semantic contribution according to the type of links and the number of children:

2 $${{\rm{w}}}_{{\rm{e}}}=1/({\rm{c}}+{\rm{nc}}({\rm{t}}))+{\rm{d}}$$

where ‘nc(t)’ is the total number of children for GO term ‘*t*’; and both ‘c’ and ‘d’ are constant parameters. The parameter ‘d’ inherited from Wang’s method refers to how strong the semantic contribution is passing through the link, which depends on the type of link between a GO term and its parent nodes. We assign ‘d’ as 0.4, 0.3, and 0.2 for ‘is-a’, ‘part-of’, and ‘regulates’, respectively. The parameter ‘c’ is subjected to the range of a valid weight (i.e. $0 < {{\rm{w}}}_{{\rm{e}}}\le 1$) based on Eq. 2, from which we can conclude that ‘c’ needs to be ≥0.67. We analyzed the performance of GOGO and the similarity between GOGO and other seven methods on different values of parameter ‘c’. This analysis was performed on 500 randomly-selected GO-term pairs with the condition that their semantic similarities based on Wang’s method are ≥0.5. Supplementary Fig. S8A shows the distribution of semantic similarities calculated by GOGO when c equals to 0.67, 1, 2, and 3, from which we can find that different c values do not cause a big difference. Supplementary Fig. S8B shows the Pearson’s correlation coefficient between GOGO and other methods when a set of ‘c’ values are applied. With the increase of ‘c’, the correlations between GOGO and other methods change slightly. In general, GOGO and other methods have the highest correlations when ‘c’ = 0.67. Therefore, we assign 0.67 to ‘c’, which is also the minimum value to make ‘w’ valid.

For each term in ${{\rm{DAG}}}_{{\rm{A}}}=({\rm{A}},\,{{\rm{T}}}_{{\rm{A}}},\,{{\rm{E}}}_{{\rm{A}}})$, it has the semantic contribution to the target term A, which is defined as the same S-value as in Wang’s method^22^:

3 $$\{\begin{array}{ll}{{\rm{S}}}_{{\rm{A}}}({\bf{t}})={\bf{1}}\, & {\bf{if}}\,{\bf{t}}={\bf{A}}\\ {{\rm{S}}}_{{\rm{A}}}({\bf{t}})=\,{\bf{\max }}\{{\rm{w}}{\rm{e}}\ast {{\rm{S}}}_{{\rm{A}}}({\rm{t}}^{\prime} )|{\rm{t}}^{\prime} \in {\bf{c}}{\bf{h}}{\bf{i}}{\bf{l}}{\bf{d}}{\bf{r}}{\bf{e}}{\bf{n}}({\bf{t}})\} & {\bf{if}}\,{\bf{t}}\ne {\bf{A}}\end{array}$$

In this way, GOGO considers the semantic contribution of ancestor terms to term A according to the number of children terms and the depth of ancestor terms, which inherit both the advantages of IC-based methods and hybrid method. The semantic value of GO term A is the summation of S-values in DAG_A_:

4 $${\rm{SV}}({\rm{A}})={\sum }_{{\rm{t}}\in {{\rm{T}}}_{{\rm{A}}}}{{\rm{S}}}_{{\rm{A}}}({\rm{t}})$$

Supplementary Table S11 shows the S-value of all GO terms in ${{\rm{DAG}}}_{{\rm{A}}}=({\rm{A}},\,{{\rm{T}}}_{{\rm{A}}},\,{{\rm{E}}}_{{\rm{A}}})$, when A is carbohydrate metabolic process GO:0005975. We also calculated the S-value of carbohydrate derivative metabolic process GO:1901135 in Supplementary Table S12. Both Supplementary Tables S11,S12 show the S-values calculated by GOGO and Wang’s method.

Formally, given ${{\rm{DAG}}}_{{\rm{A}}}=({\rm{A}},\,{{\rm{T}}}_{{\rm{A}}},\,{{\rm{E}}}_{{\rm{A}}})$ of GO term A and ${{\rm{DAG}}}_{{\rm{B}}}=({\rm{B}},\,{{\rm{T}}}_{{\rm{B}}},\,{{\rm{E}}}_{{\rm{B}}})$ of GO term B, the semantic similarity between GO term A and GO term B is defined as follows, which is the same as Wang’s method^22^:

5 $${{\rm{S}}}_{{\rm{GO}}}({\rm{A}},{\rm{B}})=\frac{{\sum }_{{\rm{t}}\in {{\rm{T}}}_{{\rm{A}}}{\cap }^{}{{\rm{T}}}_{{\rm{B}}}}({{\rm{S}}}_{{\rm{A}}}({\rm{t}})+{{\rm{S}}}_{{\rm{B}}}({\rm{t}}))}{{\rm{SV}}({\rm{A}})+{\rm{SV}}({\rm{B}})}$$

where t is the common GO terms existing in both T_A_ and T_B_; S_A_(t) and S_B_(t) are the S-values of t based on T_A_ and T_B_, respectively. Equation 5 measures the S-value through common ancestors of term A and term B normalized by the semantic values of term A and term B.

According to the example displayed in Fig. 5 and Supplementary Tables S11,12, the semantic similarity of carbohydrate metabolic process GO:0005975 and carbohydrate derivative metabolic process GO:1901135 is ${{\rm{S}}}_{{\rm{GO}}}(0005975,\,1901135)$ = 0.368.

### Functional similarity of genes

Each gene usually is annotated with multiple GO terms from various ontologies (BPO, CCO, and MFO), which means that a gene participates in multiple biological processes, has different cellular locations, or has different molecular functions. The functional similarity of genes is a combination of semantic similarities of GO terms. There are many strategies of mixing GO term semantic similarities into a gene functional similarity, such as Average (Avg)^27^, Best-Match Average (BMA)^23,28^, Average Best-Matches (ABM)^22,29^, Maximum (Max)^30^ and Best Match Maximum (BMM)^16^. Based on our evaluations (Fig. 4, Supplementary Figs S6,7), we find that BMA and ABM have the best performance among five mixing strategies. Therefore, we choose to use ABM as the default mixing strategy in GOGO. Given a gene G_1_ with m GO terms ${{\rm{go}}}_{11},\,{{\rm{go}}}_{12},\,\ldots \,{{\rm{go}}}_{1{\rm{m}}}$, the semantic similarity between another GO term go and G_1_ is defined as:

6 $${\rm{Sim}}({\rm{go}},\,{G}_{1})={}_{1\le {\rm{i}}\le {\rm{m}}}{}^{\max \,}({{\rm{S}}}_{{\rm{GO}}}({\rm{go}},\,{{\rm{go}}}_{1{\rm{i}}}))$$

where i can be any integer between 1 and m. Given a gene G_2_ with n GO terms ${{\rm{go}}}_{21},\,{{\rm{go}}}_{22},\,\ldots \,{{\rm{go}}}_{2{\rm{n}}}$, the functional similarity defined by ABM between G_1_ and G_2_ is:

7 $${\rm{Sim}}({\rm{G}}1,\,{\rm{G}}2)=\frac{{\sum }_{1\le {\rm{i}}\le {\rm{m}}}{\rm{Sim}}({{\rm{go}}}_{1{\rm{i}}},\,{{\rm{G}}}_{2})+{\sum }_{1\le {\rm{j}}\le {\rm{n}}}{\rm{Sim}}({{\rm{go}}}_{2{\rm{j}}},\,{{\rm{G}}}_{1})}{{\rm{m}}+{\rm{n}}}$$

where j can be any integer between 1 and n. Supplementary equations S1–4 are the definitions of Avg, Max, BMA, BMM, respectively. Supplementary Table S13 shows the annotated GO terms of gene PDC5 and gene PDC6 retrieved from the SGD^32^. Supplementary Table S14 shows the functional similarities between genes PDC5 and PDC6 calculated based on their GO terms in BPO.

### Parameters of the clustering algorithm

We clustered genes using the affinity propagation algorithm^33^ with the default parameters, i.e., maximum iterations 500, convits 50, and dampfact 0.95. The preference value is assigned as the median of functional similarities of gene pairs, which influences the number of clusters. In terms of the pathway “Tryptophan degradation”, we also tested larger values for the number of maximum iteration and smaller dampfact values. However, the clustering results were not affected by these changes.

### Evaluating the clustering performance using Mathew’s correlation coefficient

To evaluate the performance after adding noise genes, we manually added one noise cluster of genes to the target pathway, which only contains the randomly-selected outside genes. In other words, all outside genes are in a new cluster besides the other clusters originally existing in the target pathway. After that, we calculated true positive (TP), true negative (TN), false positive (FP), and false negative (FN) in terms of each gene in the pathway including the outside genes. For example, if the target pathway originally contains three genes: gene A, gene B, and gene C; and gene D is a newly-added outside gene, we calculate TP, TN, FP, and FN for each of the genes A, B, C, and D. For gene A, we check genes B, C, and D. If genes A and B exist in the same cluster in the original target pathway and are classified into the same cluster by GOGO or other methods, we consider this a true positive. In this way, we calculate an overall TP for gene A after looking at its relationship with genes B, C, and D. Similarly, we calculate TN, FP, and FN. Furthermore, we calculate the Matthew’s correlation coefficient (MCC)^36^ as:

8 $$MCC=\,\frac{TP\times TN-FP\times FN}{\sqrt{(TP+FP)(TP+FN)(TN+FP)(TN+FN)}}$$

The average MCC of a cluster is the average value over all of the genes in the cluster. All of the MCC scores range between [−1, 1] where 1 represents a perfect prediction; 0 represents no better than random prediction; and −1 represents total disagreement between prediction and observation. Supplementary Table S15 shows an example of calculating the MCC score for the pathway “removal of superoxide radicals”.

## Discussion

We developed an improved hybrid algorithm GOGO that calculates semantic similarities between GO terms based on GO DAG topology. We find that GO terms with higher number of children nodes in the GO DAG usually have lower IC values. Therefore, by considering the number of children nodes in the GO DAG, GOGO can mimic the property of IC. Calculating IC from a large annotation corpus usually takes a lot of computational time. GOGO does not need to calculate IC but still has the advantage of using IC.

GOGO can calculate the semantic similarities between one or more pair(s) of GO terms, functional similarities between one or more pair(s) of genes, and pairwise functional similarities between a list of genes. It can also classify multiple genes based on the functional similarities between genes. Besides the better measure of semantic similarities between GO terms, the gene clusters generated by GOGO are accurate and robust on selected SGD pathways in BPO.

The stand-alone version of GOGO contains PERL source code of the algorithms. Detailed examples of input and output files are included in the website and stand-alone package. Because GOGO and GOGO^regulates^ have very similar performances, we only release GOGO.

## Electronic supplementary material

Supplementary Information

## Data Availability

GOGO can be accessed at http://dna.cs.miami.edu/GOGO/.
