# Supplementary material for: GOGO: An improved algorithm to measure the semantic similarity between gene ontology terms
Source: Sci Rep. 2018 Oct 10;8:15107. doi: 10.1038/s41598-018-33219-y (PMC6180005; doi:10.1038/s41598-018-33219-y)
Supplement: Supplementary file 1 — Supplementary Information [file 41598_2018_33219_MOESM1_ESM.docx]

Supplementary Data for

GOGO: An Improved Algorithm to Measure the Semantic Similarity of Gene Ontology Terms

Chenguang Zhao^1^ and Zheng Wang^2,^*

^1^School of Computing, University of Southern Mississippi, 118 College Drive, Hattiesburg, MS 39406, USA

^2^Department of Computer Science, University of Miami, 1365 Memorial Drive, Coral Gables, FL 33124, USA

[*zheng.wang@miami.edu](mailto:*zheng.wang@miami.edu)_­­_

Mixing strategies of gene functional similarity:

Average:

$\mathrm{Sim}\left( G1, G2 \right)=\frac{1}{m\times n}\sum_{1\leq i\leq m, 1\leq j\leq n} \mathrm{Sim}\left( \mathrm{go}_{1i}, \mathrm{go}_{2j} \right)$ (S1)

Maximum:

$\mathrm{Sim}\left( G1, G2 \right)=\max\left\{ \mathrm{Sim}\left( \mathrm{go}_{1i}, \mathrm{go}_{2j} \right) \right|1\leq i\leq m, 1\leq j\leq n\}$ (S2)

Best-Match Average:

$\mathrm{Sim}\left( G1, G2 \right)=\frac{1}{2}\left\{ \frac{1}{m}\sum_{1\leq i\leq m} \mathrm{Sim}\left( \mathrm{go}_{1i},G_{2} \right)+\frac{1}{n}\sum_{1\leq j\leq n} \mathrm{Sim}\left( \mathrm{go}_{2j},G_{1} \right) \right\}$ (S3)

Best Match Maximum:

$\mathrm{Sim}\left( G1, G2 \right)=max\left\{ \frac{1}{m}\sum_{1\leq i\leq m} \mathrm{Sim}\left( \mathrm{go}_{1i},G_{2} \right), \frac{1}{n}\sum_{1\leq j\leq n} \mathrm{Sim}\left( \mathrm{go}_{2j},G_{1} \right) \right\}$ (S4)

Figure S1. The mevalonate pathway retrieved from the SGD database.


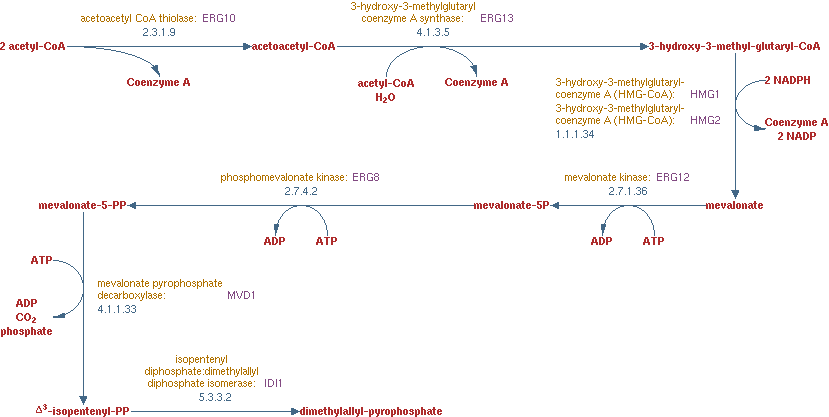


Figure S2. The phenylalanine degradation pathway retrieved from the SGD database.





Figure S3. The removal of superoxide radicals pathway retrieved from the SGD database.


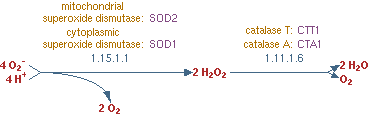


Figure S4. The valine degradation pathway retrieved from the SGD database.


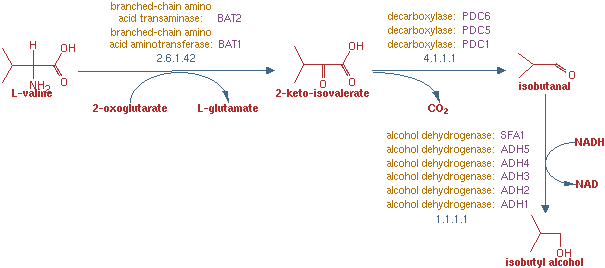


Figure S5. The mannose degradation pathway retrieved from the SGD database.


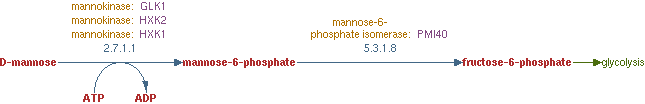


Figure S6. Violin and box plots of average MCC scores on pathways in CCO. Figure S6a shows the MCC scores calculated when no outside genes are added i.e., only using the genes originally existent in the target pathways. Figure S6b shows the MCC scores after randomly-selected outside genes are added. Figure S6c shows the MCC scores after randomly-selected outside genes from the same EC category are added, i.e., the first two EC numbers are the same.


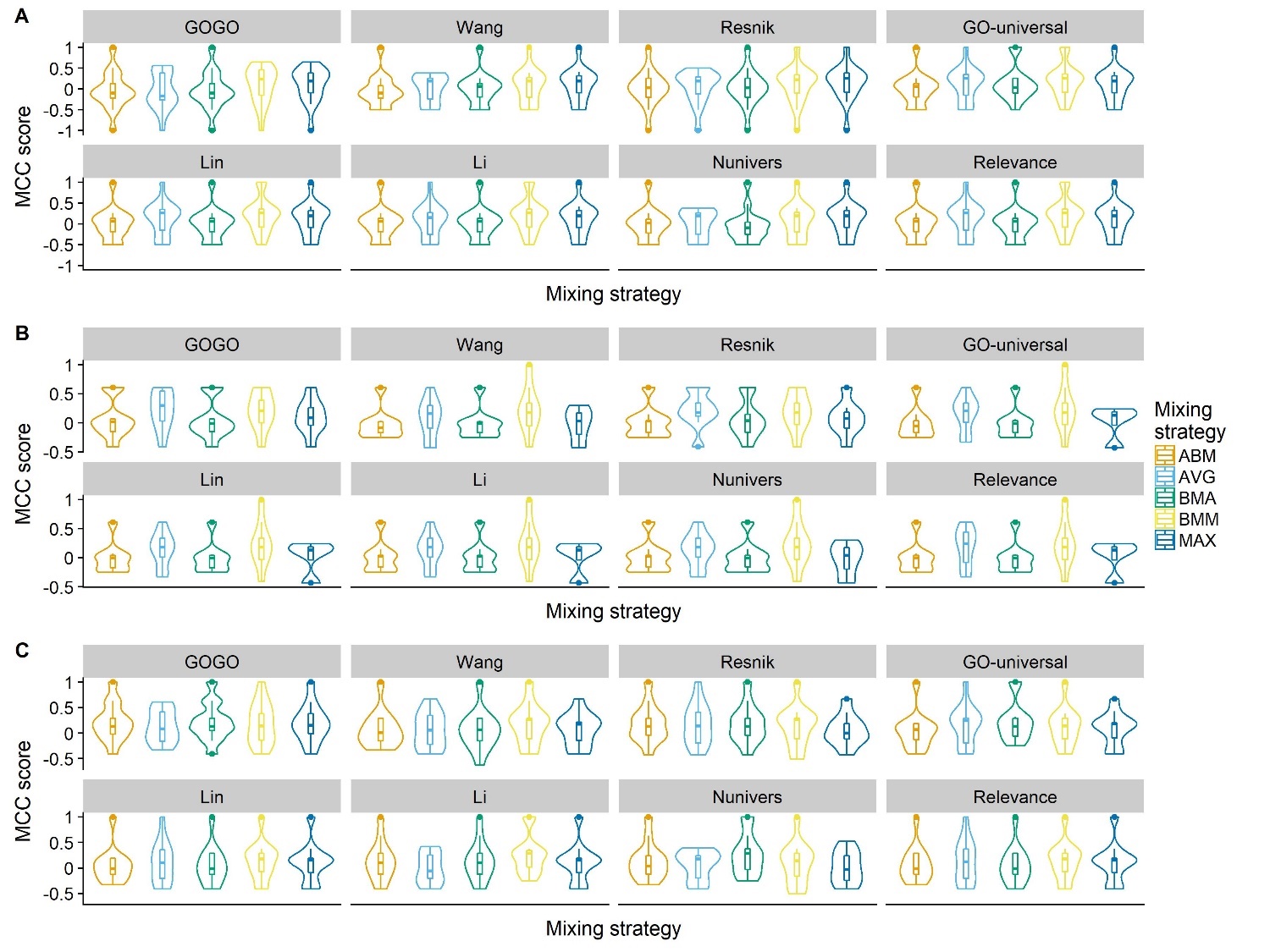


Figure S7. Violin and box plots of average MCC scores on pathways in MFO. Figure S7a shows the MCC scores calculated when no outside genes are added i.e., only using the genes originally existent in the target pathways. Figure S7b shows the MCC scores after randomly-selected outside genes are added. Figure S7c shows the MCC scores after randomly-selected outside genes from the same EC category are added, i.e., the first two EC numbers are the same.


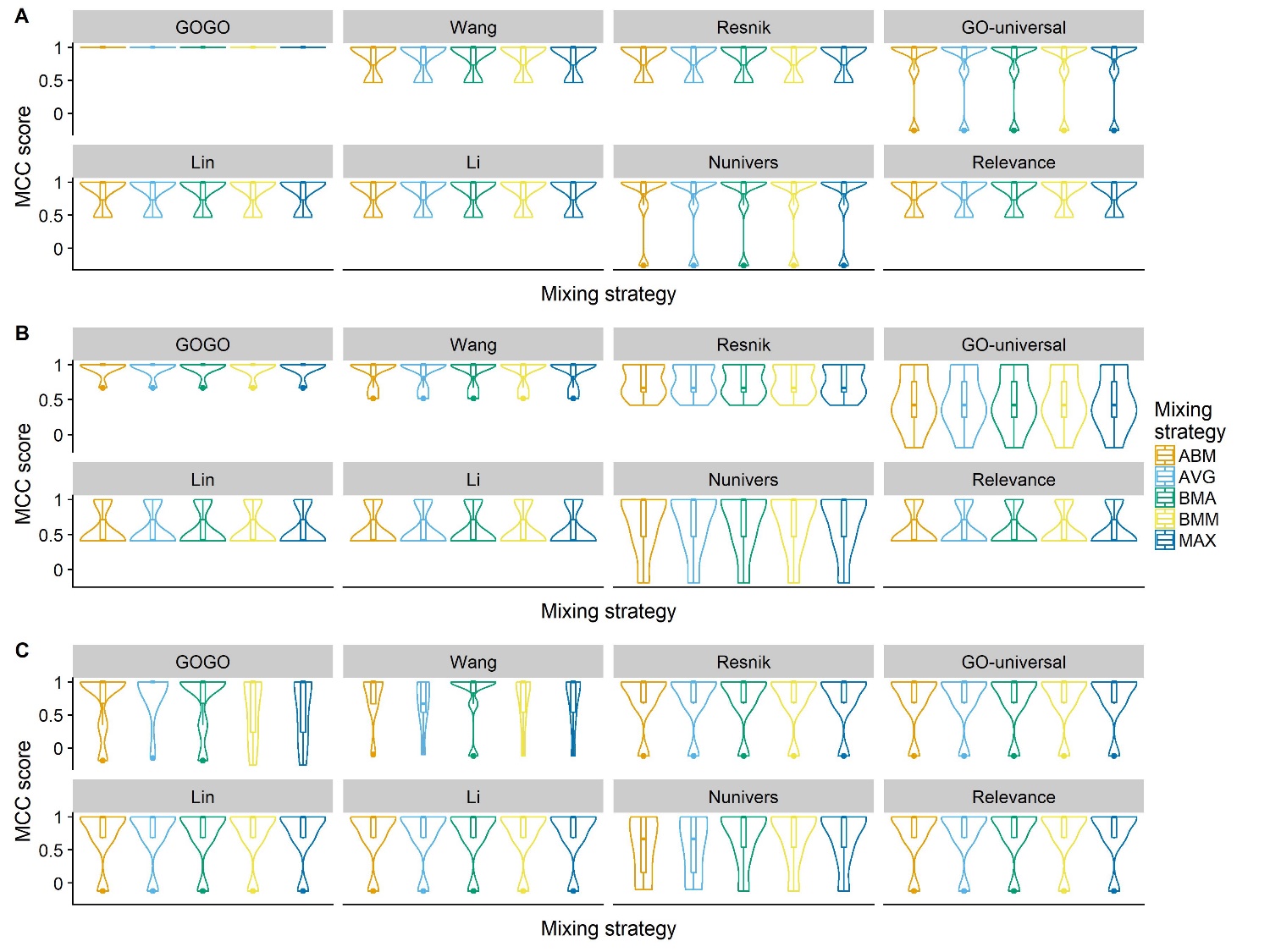


Figure S8. The performance of GOGO on different values of parameter ‘c’. Figure 8a is the distribution of semantic similarity of GO terms when c equals to 0.67, 1, 2, and 3. Figure 8b is the Pearson’s correlations coefficient between GOGO and other methods at different values of parameter ‘c’. This analysis is performed on 500 randomly-selected GO-term pairs with semantic similarities of Wang’s method ≥ 0.5.


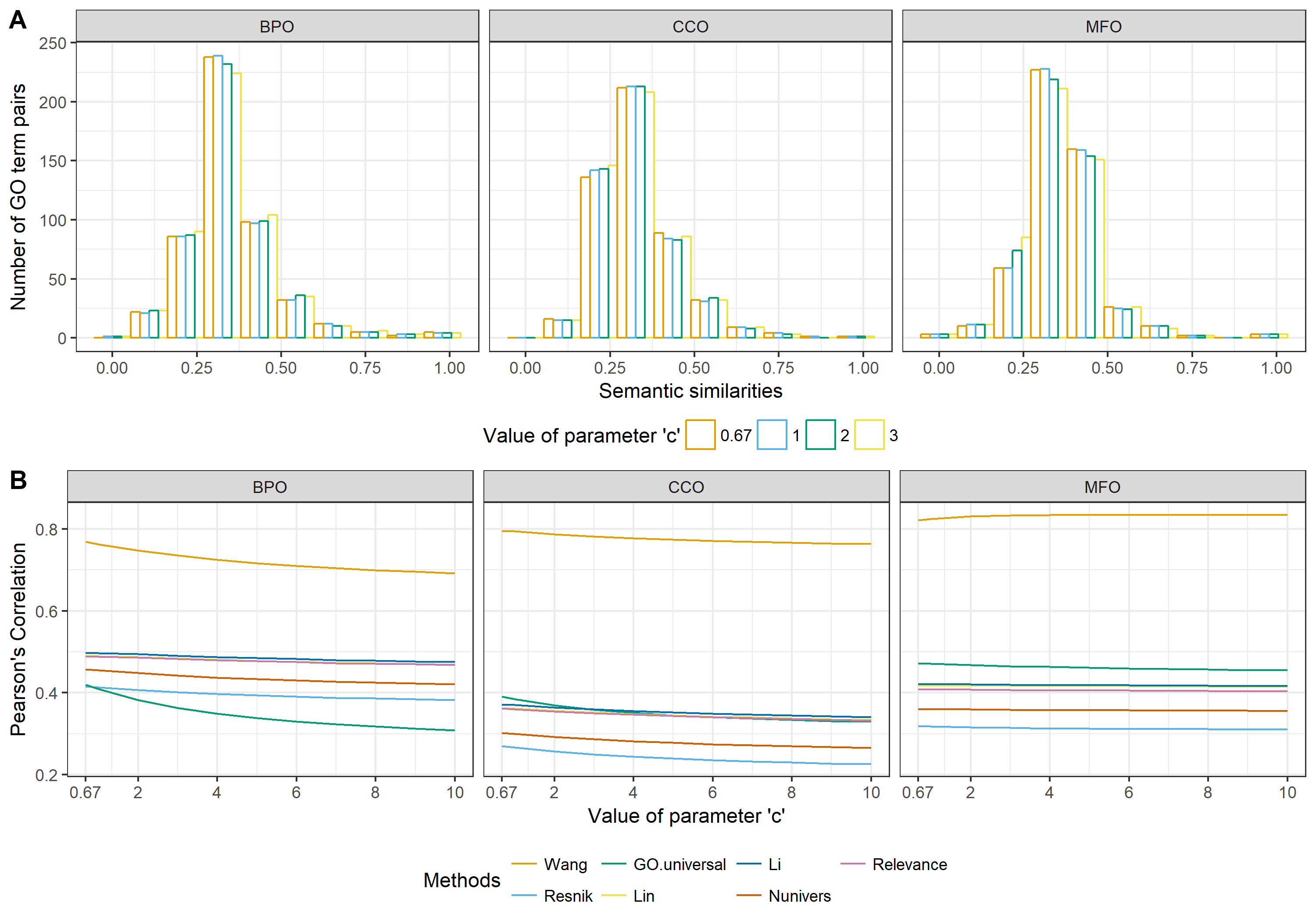


Table S1. Pearson’s correlation coefficient matrices between GOGO and other methods in BPO, CCO, and MFO. For each gene ontology, Pearson’s correlation coefficient is generated based on 500 randomly-selected GO-term pairs without threshold.

| BPO | GOGO | Wang et al. | Resnik | GO-universal | Lin | Li et al. | Nunivers | Relevance |
| --- | --- | --- | --- | --- | --- | --- | --- | --- |
| GOGO | 1.00 | 0.93 | 0.72 | 0.77 | 0.72 | 0.74 | 0.71 | 0.72 |
| Wang et al. |  | 1.00 | 0.81 | 0.80 | 0.82 | 0.82 | 0.81 | 0.82 |
| Resnik |  |  | 1.00 | 0.61 | 0.99 | 0.99 | 0.99 | 0.99 |
| GO-universal |  |  |  | 1.00 | 0.59 | 0.60 | 0.60 | 0.59 |
| Lin |  |  |  |  | 1.00 | 1.00 | 1.00 | 1.00 |
| Li et al. |  |  |  |  |  | 1.00 | 0.99 | 1.00 |
| Nunivers |  |  |  |  |  |  | 1.00 | 1.00 |
| Relevance |  |  |  |  |  |  |  | 1.00 |
| CCO | GOGO | Wang et al. | Resnik | GO-universal | Lin | Li et al. | Nunivers | Relevance |
| GOGO | 1.00 | 0.90 | 0.70 | 0.67 | 0.73 | 0.72 | 0.71 | 0.72 |
| Wang et al. |  | 1.00 | 0.80 | 0.74 | 0.80 | 0.78 | 0.79 | 0.79 |
| Resnik |  |  | 1.00 | 0.48 | 0.97 | 0.97 | 0.97 | 0.97 |
| GO-universal |  |  |  | 1.00 | 0.49 | 0.48 | 0.48 | 0.48 |
| Lin |  |  |  |  | 1.00 | 1.00 | 0.99 | 1.00 |
| Li et al. |  |  |  |  |  | 1.00 | 0.99 | 1.00 |
| Nunivers |  |  |  |  |  |  | 1.00 | 0.99 |
| Relevance |  |  |  |  |  |  |  | 1.00 |
| MFO | GOGO | Wang et al. | Resnik | GO-universal | Lin | Li et al. | Nunivers | Relevance |
| GOGO | 1.00 | 0.87 | 0.68 | 0.65 | 0.73 | 0.74 | 0.70 | 0.73 |
| Wang et al. |  | 1.00 | 0.82 | 0.88 | 0.84 | 0.81 | 0.82 | 0.82 |
| Resnik |  |  | 1.00 | 0.81 | 0.97 | 0.97 | 0.98 | 0.97 |
| GO-universal |  |  |  | 1.00 | 0.79 | 0.75 | 0.78 | 0.77 |
| Lin |  |  |  |  | 1.00 | 0.99 | 0.99 | 0.99 |
| Li et al. |  |  |  |  |  | 1.00 | 0.98 | 1.00 |
| Nunivers |  |  |  |  |  |  | 1.00 | 0.99 |
| Relevance |  |  |  |  |  |  |  | 1.00 |

Table S2. Similarity table of genes set of tryptophan degradation measured by GOGO in BPO

| GOGO | ARO9 | ARO8 | ARO10 | PDC6 | PDC5 | PDC1 | SFA1 | ADH5 | ADH4 | ADH3 | ADH2 | ADH1 |
| --- | --- | --- | --- | --- | --- | --- | --- | --- | --- | --- | --- | --- |
| ARO9 |  | 1.000 | 0.353 | 0.360 | 0.368 | 0.368 | 0.323 | 0.322 | 0.265 | 0.351 | 0.299 | 0.322 |
| ARO8 |  |  | 0.353 | 0.360 | 0.368 | 0.368 | 0.323 | 0.322 | 0.265 | 0.351 | 0.299 | 0.322 |
| ARO10 |  |  |  | 0.791 | 0.736 | 0.736 | 0.525 | 0.503 | 0.543 | 0.542 | 0.494 | 0.503 |
| PDC6 |  |  |  |  | 0.749 | 0.749 | 0.406 | 0.396 | 0.410 | 0.407 | 0.612 | 0.396 |
| PDC5 |  |  |  |  |  | 1.000 | 0.392 | 0.547 | 0.432 | 0.404 | 0.392 | 0.547 |
| PDC1 |  |  |  |  |  |  | 0.392 | 0.547 | 0.432 | 0.404 | 0.392 | 0.547 |
| SFA1 |  |  |  |  |  |  |  | 0.524 | 0.566 | 0.587 | 0.516 | 0.524 |
| ADH5 |  |  |  |  |  |  |  |  | 0.574 | 0.847 | 0.748 | 1.000 |
| ADH4 |  |  |  |  |  |  |  |  |  | 0.642 | 0.546 | 0.574 |
| ADH3 |  |  |  |  |  |  |  |  |  |  | 0.832 | 0.847 |
| ADH2 |  |  |  |  |  |  |  |  |  |  |  | 0.748 |
| ADH1 |  |  |  |  |  |  |  |  |  |  |  |  |

Table S3. Similarity table of genes set of tryptophan degradation measured by Wang’s method in BPO

| Wang | ARO9 | ARO8 | ARO10 | PDC6 | PDC5 | PDC1 | SFA1 | ADH5 | ADH4 | ADH3 | ADH2 | ADH1 |
| --- | --- | --- | --- | --- | --- | --- | --- | --- | --- | --- | --- | --- |
| ARO9 |  | 1.000 | 0.544 | 0.532 | 0.546 | 0.546 | 0.485 | 0.473 | 0.470 | 0.516 | 0.461 | 0.461 |
| ARO81 |  |  | 0.544 | 0.532 | 0.546 | 0.546 | 0.485 | 0.473 | 0.470 | 0.516 | 0.461 | 0.473 |
| ARO10 |  |  |  | 0.845 | 0.805 | 0.805 | 0.663 | 0.653 | 0.679 | 0.684 | 0.640 | 0.653 |
| PDC6 |  |  |  |  | 0.778 | 0.778 | 0.556 | 0.544 | 0.562 | 0.568 | 0.726 | 0.544 |
| PDC5 |  |  |  |  |  | 1.000 | 0.559 | 0.687 | 0.562 | 0.592 | 0.562 | 0.687 |
| PDC1 |  |  |  |  |  |  | 0.559 | 0.687 | 0.562 | 0.592 | 0.562 | 0.687 |
| SFA1 |  |  |  |  |  |  |  | 0.643 | 0.695 | 0.687 | 0.629 | 0.643 |
| ADH5 |  |  |  |  |  |  |  |  | 0.653 | 0.896 | 0.800 | 1.000 |
| ADH4 |  |  |  |  |  |  |  |  |  | 0.711 | 0.633 | 0.653 |
| ADH3 |  |  |  |  |  |  |  |  |  |  | 0.864 | 0.896 |
| ADH2 |  |  |  |  |  |  |  |  |  |  |  | 0.800 |
| ADH1 |  |  |  |  |  |  |  |  |  |  |  |  |

Table S4. Similarity table of genes set of tryptophan degradation measured by Resnik’s method in BPO

| Resnik | ARO9 | ARO8 | ARO10 | PDC6 | PDC5 | PDC1 | SFA1 | ADH5 | ADH4 | ADH3 | ADH2 | ADH1 |
| --- | --- | --- | --- | --- | --- | --- | --- | --- | --- | --- | --- | --- |
| ARO9 |  | 1.000 | 0.345 | 0.360 | 0.352 | 0.352 | 0.214 | 0.239 | 0.227 | 0.239 | 0.222 | 0.222 |
| ARO8 |  |  | 0.345 | 0.360 | 0.352 | 0.352 | 0.214 | 0.239 | 0.227 | 0.239 | 0.222 | 0.239 |
| ARO10 |  |  |  | 0.840 | 0.785 | 0.785 | 0.545 | 0.565 | 0.563 | 0.567 | 0.552 | 0.565 |
| PDC6 |  |  |  |  | 0.828 | 0.828 | 0.481 | 0.541 | 0.495 | 0.500 | 0.654 | 0.541 |
| PDC5 |  |  |  |  |  | 1.000 | 0.451 | 0.647 | 0.547 | 0.580 | 0.583 | 0.647 |
| PDC1 |  |  |  |  |  |  | 0.451 | 0.647 | 0.547 | 0.580 | 0.583 | 0.647 |
| SFA1 |  |  |  |  |  |  |  | 0.549 | 0.543 | 0.550 | 0.529 | 0.549 |
| ADH5 |  |  |  |  |  |  |  |  | 0.695 | 0.943 | 0.887 | 1.000 |
| ADH4 |  |  |  |  |  |  |  |  |  | 0.636 | 0.594 | 0.695 |
| ADH3 |  |  |  |  |  |  |  |  |  |  | 0.885 | 0.943 |
| ADH2 |  |  |  |  |  |  |  |  |  |  |  | 0.887 |
| ADH1 |  |  |  |  |  |  |  |  |  |  |  |  |

Table S5. Clustering results comparison of genes in mevalonate pathway

|  | GOGO | GOGO^regulates^ | Wang | Resnik | SGD |
| --- | --- | --- | --- | --- | --- |
| MFO | ERG10 ERG13 | ERG10 ERG13 | ERG10 ERG13 | ERG10 ERG13 | ERG10 ERG13 |
|  | HMG2 HMG1 | HMG2 HMG1 | HMG2 HMG1  IDI1 MVD1 | HMG2 HMG1 | HMG2 HMG1 |
|  | ERG8 ERG12 | ERG8 ERG12 | ERG8 ERG12 | ERG8 ERG12  MVD1 IDI1 | ERG8 ERG12 |
|  | MVD1 IDI1 | MVD1 IDI1 |  |  | MVD1 IDI1 |

Table S6. Clustering results comparison of genes in phenylalanine degradation

|  | GOGO | GOGO^regulates^ | Wang | Resnik | SGD |
| --- | --- | --- | --- | --- | --- |
| BPO | ARO8 ARO9 | ARO8 ARO9 | ARO8 ARO9 | ARO8 ARO9 | ARO8 ARO9 |
|  | PDC6 PDC1 PDC5 ARO10 | PDC6 PDC1 PDC5 ARO10 | ARO10 ADH4 PDC6 SFA1 | ARO10 PDC1 PDC5 PDC6 | PDC6 PDC1 PDC5 ARO10 |
|  |  |  |  | SFA1 |  |
|  | ADH1 ADH4 SFA1 ADH3 ADH2 ADH5 | ADH3 ADH5 ADH1 | ADH3 ADH5 ADH1 | ADH3 ADH4 ADH2 ADH5 ADH1 | ADH1 ADH4 SFA1 ADH3 ADH2 ADH5 |
|  |  |  | PDC1 PDC5 |  |  |
|  |  |  | ADH2 |  |  |

Table S7. Clustering results comparison of genes in removal of superoxide radicals

|  | GOGO | GOGO^regulates^ | Wang | Resnik | SGD |
| --- | --- | --- | --- | --- | --- |
| BPO | CTT1 CTA1 | CTT1 CTA1 | SOD1 | SOD1 | CTT1 CTA1 |
|  | SOD2 SOD1 | SOD2 SOD1 | SOD2 CTT1 CTA1 | SOD2 CTT1 CTA1 | SOD2 SOD1 |
| MFO | CTT1 CTA1 | CTT1 CTA1 | CTT1 CTA1 | CTT1 CTA1 | CTT1 CTA1 |
|  | SOD2 SOD1 | SOD2 SOD1 | SOD2 SOD1 | SOD2 SOD1 | SOD2 SOD1 |

Table S8. Clustering results comparison of genes in valine degradation

|  | GOGO | GOGO^regulates^ | Wang | Resnik | SGD |
| --- | --- | --- | --- | --- | --- |
| BPO | PDC6 PDC1 PDC5 | PDC6 PDC1 PDC5 | PDC5 PDC1 | SFA1 | PDC6 PDC1 PDC5 |
|  |  |  | ADH4 ADH2 PDC6 | PDC1 PDC5 PDC6 |  |
|  | BAT1 BAT2 | BAT1 BAT2 | BAT1 BAT2 | BAT2 BAT1 | BAT1 BAT2 |
|  | ADH1 ADH4 SFA1 ADH3 ADH2 ADH5 | ADH1 ADH4 SFA1 ADH3 ADH2 ADH5 | ADH3 ADH5 SFA1 ADH1 | ADH3 ADH4 ADH2 ADH5 ADH1 | ADH1 ADH4 SFA1 ADH3 ADH2 ADH5 |

Table S9. Clustering results comparison of genes in mannose degradation

|  | GOGO | GOGO^regulates^ | Wang | Resnik | SGD |
| --- | --- | --- | --- | --- | --- |
| MFO | HXK2 GLK1 HXK1 | HXK2 GLK1 HXK1 | HXK2 GLK1 HXK1 | HXK2 GLK1 HXK1 | HXK2 GLK1 HXK1 |
|  | PMI40 | PMI40 | PMI40 | PMI40 | PMI40 |

Table S10. Running times of different methods. The calculation time of semantic similarities is measured by calculating 100 randomly selected GO term pairs in BPO. Pre-calculation of IC-based method costs 3,781 seconds when UniProt is used as the annotation corpus. All jobs are based on one CPU: Intel(R) Xeon(R) CPU E5-2650 v4 @ 2.20GHz.

|  | GOGO | Wang | Resnik | Lin | Li | Nunivers | Relevance |
| --- | --- | --- | --- | --- | --- | --- | --- |
| Time(second) | 0.784 | 2.95 | 1.11 | 1.19 | 1.18 | 1.26 | 1.25 |

Table S11. Weights for the edge from children nodes and S-values for carbohydrate metabolic process GO:0005975 and its ancestor terms, with both GOGO and Wang’s method.

| GO terms | 0005975 | 0044238 | 0071704 | 0008152 | 0008150 |
| --- | --- | --- | --- | --- | --- |
| $\mathbf{w}_{\mathbf{e}}$ (GOGO) | Irrelevant | 1 / (0.67 + 6) + 0.4 = 0.55 | 1 / (0.67 + 53) + 0.4 = 0.419 | 1 / (0.67 + 15) + 0.4 = 0.464 | 1 / (0.67 + 24) + 0.4 = 0.441 |
| S-value (GOGO) | 1 | 0.550 | 0.419 | 0.255 | 0.112 |
| $\mathbf{w}_{\mathbf{e}}$ (Wang) | Irrelevant | 0.8 | 0.8 | 0.8 | 0.8 |
| S-value (Wang) | 1 | 0.8 | 0.8 | 0.64 | 0.512 |
| # of children | Irrelevant | 6 | 53 | 15 | 24 |

Table S12. Weights for the edge from children nodes and S-values for carbohydrate derivative metabolic process GO:1901135 and its ancestor terms, with both GOGO and Wang’s method.

| GO terms | 1901135 | 0071704 | 0008152 | 0008150 |
| --- | --- | --- | --- | --- |
| $\mathbf{w}_{\mathbf{e}}$ (GOGO) | Irrelevant | 1 / (0.67 + 53) + 0.4 = 0.419 | 1 / (0.67 + 15) + 0.4 = 0.464 | 1 / (0.67 + 24) + 0.4 = 0.441 |
| S-value (GOGO) | 1 | 0.419 | 0.194 | 0.086 |
| $\mathbf{w}_{\mathbf{e}}$ (Wang) | Irrelevant | 0.8 | 0.8 | 0.8 |
| S-value (Wang) | 1 | 0.8 | 0.64 | 0.512 |
| # of children | Irrelevant | 53 | 15 | 24 |

Table S13. Genes PDC5 and PDC6 and annotated GO terms in BPO.

| PDC5 | |
| --- | --- |
| GO:0019655 | glycolytic fermentation to ethanol |
| GO:0006090 | pyruvate metabolic process |
| GO:0000949 | aromatic amino acid family catabolic process to alcohol via Ehrlich pathway |
| GO:0006569 | tryptophan catabolic process |
| GO:0006559 | L-phenylalanine catabolic process |
| PDC6 | |
| GO:0006067 | ethanol metabolic process |
| GO:0000949 | aromatic amino acid family catabolic process to alcohol via Ehrlich pathway |
| GO:0006569 | tryptophan catabolic process |
| GO:0006559 | L-phenylalanine catabolic process |

Table S14. Functional similarities between genes PDC5 and PDC6 calculated based on their GO terms in BPO. Based on the equation (7) in the manuscript, the functional similarity given by GOGO between genes PDC5 and PDC6 is (0.291 + 0.155 + 1 + 1 + 1 + 0.291 + 1 + 1 + 1) / (5 + 4) = 0.749.

| PDC5  PDC6 | GO:0019655 | GO:0006090 | GO:0000949 | GO:0006569 | GO:0006559 |
| --- | --- | --- | --- | --- | --- |
| GO:0006067 | 0.291 | 0.073 | 0.105 | 0.036 | 0.070 |
| GO:0000949 | 0.142 | 0.116 | 1 | 0.397 | 0.408 |
| GO:0006569 | 0.103 | 0.056 | 0.397 | 1 | 0.185 |
| GO:0006559 | 0.114 | 0.155 | 0.408 | 0.185 | 1 |

Table S15. MCC of cluster calculation table for pathway “removal of superoxide radicals”. There are two clusters for each true and predicted condition, i.e. C1 and C2 for true condition; C1’ and C2’ for predicted condition. CTA1, CTT1, SOD2, and SOD1 are gene names. The nodes of genes are filled in line by line. Take the line of CTA1 of C1’ in predicted condition for example, CTA1 is in the same cluster as CTT1, which is same as true condition. Thus, the node of CTA1 and CTT1 is true positive (TP). CTA1 and SOD2 are not in the same cluster but are clustered into the same cluster C1’. Therefore, the node of CTA1 and SOD2 is false positive (FP). Etc. MCC of genes are calculated based on each column of genes by Formula 8 in main manuscript. The MCC of cluster is the average of MCC of genes.

| Note: ‘C’ refers to Cluster, ‘NA’ refers to gene’s MCC does not exist. | | | True condition | | | |
| --- | --- | --- | --- | --- | --- | --- |
|  |  |  | C 1 | | C 2 | |
|  |  |  | CTA1 | CTT1 | SOD2 | SOD1 |
| Predicted condition | C 1’ | CTA1 |  | TP | FP | TN |
|  |  | CTT1 | TP |  | FP | TN |
|  |  | SOD2 | FP | FP |  | FN |
|  | C 2’ | SOD1 | TN | TN | FN |  |
| MCC of gene | | | 0.5 | 0.5 | -1 | NA |
| MCC of cluster | | | 0.5 | | -1 | |
